# The evaluation of chosen body posture parameters in children with scoliosis – own materials

**DOI:** 10.1186/1748-7161-9-S1-P13

**Published:** 2014-12-04

**Authors:** Zbigniew Śliwiński, Wojciech Kufel, Bartłomiej Halat, Beata Michalak, Danuta Śliwińska, Grzegorz Śliwiński

**Affiliations:** 1 Physiotherapy Center Zgorzelec, Zgorzelec, Poland; 2 Dresden University of Technology - Institute of Biomedical Engineering, Dresden, Germany

## Introduction

Treatment of children with scoliosis requires the monitoring and evaluation of posture parameters. The raster stereographic method can be used at any time to observe the therapy progress due to its harmlessness towards the child.

## Material and methods

We evaluated 140 children with idiopathic scoliosis treated by the FED method at the Rehabilitation Centre in Zgorzelec. The children were assessed by the 4D DIERS Formetric III at the beginning and the end of the month of the stay. Some of the patients were evaluated six-months after the FED treatment. One of the parameters selected to assess the therapy outcome is the rotation of the trunk area beginning at the apex deriving from radiography.

## Results

Preliminary results in both groups, the group assessed after a month and the group assessed after six-months, reveal a reduction of the rotation parameter by an average of 40.

## Conclusions

After a preliminary analysis of the trunk posture in children with idiopathic scoliosis assessed by DIERS Formetric III 4D, the raster stereographic method can be considered a fast, secure way to follow-up the outcome of the FED therapy.

